# Amifostine otoprotection to cisplatin ototoxicity: a guinea pig study using otoacoustic emission distortion products (DPOEA) and scanning electron microscopy

**DOI:** 10.1016/S1808-8694(15)31322-7

**Published:** 2015-10-20

**Authors:** Miguel Angelo Hyppolito, José Antonio A. de Oliveira, Ricardo Miranda Lessa, Maria Rossato

**Affiliations:** ^1^ Ph.D. in Medicine, Professor, FMRP - USP (assistant physician); ^2^ Faculty Professor, Coordinator of the Division of Otorhinolaryngology, Department of Ophthalmology, Otorhinolaryngology and Head and Neck Surgery, FMRP-USP; ^3^ Master, Post-graduation, Division of Otorhinolaryngology, Department of Ophthalmology, Otorhinolaryngology and Head and Neck Surgery, FMRP-USP; ^4^ Laboratory technician

**Keywords:** cisplatin, otoprotective agents, amifostine

## Abstract

**C**isplatin is an antineoplastic drug for cancer treatment in children and adults. The side effects of cisplatin ototoxicity are significant: irreversible bilateral hearing damage to high frequencies (4 kHz - 8 kHz). Reports recognize some drugs that are associated with cisplatin to obtain an otoprotector effect. The ototoxicity mechanisms of cisplatin are related to injury of hair cell oxidation mechanism, especially of outer hair cells. **Aim**: Using otoacoustic emissions distortion products (DPOEA) and scanning electron microscopy we intended to verify the action of amifostine, a radioprotective drug that has well known antioxidant characteristics and otoprotector effects to cisplatin injury. **Study design**: Experimental. **Material and Method**: We used an experimental guinea pig model. The study was performed as follows: group 1: 6 animals, 12 ears, cisplatin 8.0 mg/Kg/day (IP), 3 days. Group 2: 6 animals, 12 ears, amifostine 100 mg/Kg/day (IP) and after 90 minutes, cisplatin 8.0 mg/Kg/day (IP), 3 days and group 3: 3 animals, 6 ears, amifostine 100 mg/Kg/day (IP), 3 days. **Results**: DPOEA were present before and after treatment in groups 2 and 3. The normal cilium architecture of outer hair cells was supported in all cochlear turns in groups 2 and 3. We concluded that amifostine has a potential otoprotector effect against cisplatin ototoxicity and could be used in clinical trials.

## INTRODUCTION

The improvement in survival of patients with cancer by using more effective antineoplastic drugs, such as cisplatin, has favored the increase in incidence of side effects, especially in the central nervous system, kidneys and hearing system.^1^

Thus, different substances have been studied to try to protect subjects from these effects, without reducing antitumor activity of cisplatin.

Cisplatin causes bilateral symmetrical sensorineural loss in frequencies of 4 to 8kHz, with association of tinnitus^2^.

The incidence of ototoxicity is increased as the accumulated dose of cisplatin is over 200mg/m2 of body surface. As a result of high frequency audiometry studies, the incidence of hearing loss has increased and reached up to 70% for frequencies up to 16kHz^3^.

McAlpine and Johnstone, 1990, in an experimental study in guinea pigs observed damage to outer hair cells on the basal turn of the cochlea, without damage to stria vascularis^4^.

Studies conducted in the 90's had shown that cisplatin inhibited the activity of adenylcyclase in stria vascularis, inhibited DNA and RNA, protein synthesis and increased levels of oxygen free radicals, which are toxic to the cell.^5^

Ravi, 1995, showed that the cochlea might suffer affections to its anti-oxidative capacity, with reduction of cochlear glutation levels, reduction of glutation oxidase and increase in activity of catalase and dismutase superoxide enzymes.^6^

Different otoprotective drugs have been tested, and most of them act as cell free anti-radical, among which we can include amifostine (WR 2721, acid S - 2[3-aminopropylamine] ethylphosphorothiol), developed by Walter Red Army Institute, in the 50's, to protect the toxic effects of radiotherapy, without affecting the antitumoral potential.7., 8.

Yuhas and Culo, 1980, were the first ones to show that amifostine promoted a reduction in nephrotoxicity induced by cisplatin, without affecting antitumor activity, which was later confirmed by the studies conducted by Glover et al., 1986 and 1987.7., 9., 10.

Mollman et al., 1988, showed slight reduction in ototoxicity of cisplatin in patients previously treated with amifostine^11^. Rubin et al., 1995, showed that there was no ototoxicity in speech frequencies in any of the patients treated with amifostine and cisplatin^12^.

Amifostine is converted into an active sulphidic compound, named WR1065, which acts as a cytoprotector, chelating free radicals. There is selective protection of amifostine to normal tissue cells, owing to high concentration of this compound in tumor cells. This fact is explained by reduction of alkaline phosphatase activity in tumor cells, low tumor vascularization and tumor anaerobe metabolism that causes a with very low pH medium, which does not allow intracellular entry of WR1065 because it requires a ph between 6.6 and 8.2.7., 8.

In the 80's, amifostine was approved by FDA to be used in patients that received cisplatin to prevent cisplatin nephrotoxicity^13^. However, its use is not recommended in cases of potentially curable tumors because the influence of cisplatin in the efficacy of chemotherapy is not exactly known yet12., 14.

We did not find any experimental study in the literature that had confirmed outer hair cells' protection by amifostine to the toxic effects of cisplatin.

Two experimental studies in hamsters using surface electron microscopy and brainstem evoked potential have tested the otoprotection potential of amifostine and other drugs to the effects of cisplatin. They showed that sodium thiosulfate and diethyldithiocarbamate are more effective as otoprotectors than amifostine and fosfomycin15., 16..

We decided to study the otoprotective effects of amifostine to damage to outer hair cells caused by cisplatin, using functional measures through otoacoustic emission distortion product and anatomical assessment of damage caused to outer hair cells by electron scanning microscopy.

## MATERIAL AND METHODS

### Selection of Experimental Animals

The experimental animal selected was albino guinea pigs since they are easy to handle and the cochlea is easy to dissect, plus it is easy to infuse anesthetic drugs and experimental drugs through intraperitoneal access and they are more sensible to the effects of cisplatin. The required dose is 8.0 mg/Kg/day for three consecutive days, which leads to significant cochlear affections.

Guinea pigs allow appropriate maintenance and compliance with the guidelines of care and use of laboratory animals of Institute of Laboratory Animal Resources, Commission on Life Sciences, National Research Council, National Academy Press, Washington, DC. (1996).

Animals were selected at the Central Animal Laboratory, University of Sao Paulo - Campus of Ribeirão Preto, by studying Preyer' reflex. We chose animals weighting on average 400 and 600 grams, because they were animals resistant to systemic side effects of cisplatin, with lack of appetite, weight loss, dehydration and diarrhea.17., 18.

After 24-hour hearing rest, animals were reassessed and we conducted manual otoscopy. Animals that presented signals of external or acute otitis media, difficult to remove cerumen, inflammatory affections of external auditory canal, or even very narrow canal to appropriately accommodate the probe of the otoacoustic emissions, were excluded from the study and those that presented easy to remove cerumen were maintained.

Guinea pigs were submitted to hearing screening by DPOAE in sound-proof booth and under anesthesia with Ketamine (65 mg/Kg) and Xylazin (6.5 mg/Kg). The ones that presented DPOAE in at least one of the ears were selected for the experiment.

Animals were maintained in the Animal Laboratory of the Experimental Surgery, Department of Surgery, Medical School, Ribeirão Preto-USP.

Given that it was an experiment using systemic drugs, for each tested animal we considered two cochleae and animals that presented OAE in one ear were also used.

### Drugs Used, Doses and Administration Route

1. Cisplatin (10 mg/ml) - 0.75; 1.5 and 8.0 mg/Kg/day intraperitoneal access;
2. Xylazin (2g/100ml) - 6.5mg/Kg intraperitoneal access;
3. Ketamine Chlorhydrate (50 mg/ml) - 65mg/Kg - intraperitoneal access;
4. Amifostine 100 mg/Kg/day - intraperitoneal access.

For controlled application of tested drugs, we used 1cc disposable syringes for each animal. For intraperitoneal application, we used disposable BD syringes size 21G1 (25 X 8 - 0.8 × 25 millimeters).

### Studied Groups

**Table:** 

Group 1:	06 animals - 12 ears - cisplatin dose of 8.0 mg/Kg/day, intraperitoneal access, for three days.
Group 2:	06 animals - 12 ears - Amifostine dose of 100 mg/Kg/day, intraperitoneal access and 90 minutes after, cisplatin 8.0 mg/Kg/day, intraperitoneal access for three days.
Group 3:	03 animals - 06 ears - Amifostine dose of 100 mg/Kg/day, intraperitoneal access for three days.

### Auditory Functional Assessment

 

### Distortion product otoacoustic emissions

#### Equipment: ILO 92 CAE System Otodynamics LTD

Guinea pigs were anesthetized with Ketamine Chlorhydrate and Xylazin before undergoing the tests. Before OAE, they were submitted to manual otoscopy to assess the external auditory canal and tympanic membrane, and those with signals of otitis or difficult to remove cerumen were excluded.

DPOAE was conducted before treatment and some minutes before the animals were sacrificed, following the frequency 2F1 - F2 with the ratio F1:F2 = 1.22, resolution of two points per octave.

We considered OAE as of the frequency of 1.5 kHz, because the dimensions of the external auditory canal of guinea pigs result in difficulty to detect OAE below this frequency, and the responses coincide with responses to noise.

Thus, we analyzed the frequency of 2kHz, we provided one pure tone above and another one below it, so as the relation between them was 1.22, automatically reaching the frequency response resulting from the relation 2F1-F2 (below the assessed frequency) and 2F2-F1 (above the resulting frequency). We should also bear in mind that intensities F2 and F1 can be either similar or different. In the present study, we used intensities that were similar to 70dB SPL. The intensity of the triggering stimuli may vary from 0 to 70 dB SPL and it can be measured in the range 500 to 8000Hz.^19^

Resulting otoacoustic emissions are normally about 55 dB SPL less loud than the triggering stimulus. With 70 dB SPL, the generated DPOAE would range approximately from 10 to 15 dB SPL^20^.

Thus, we detected the DP gram, or audiocochleogram, in which the stimulus is a sound and a response that is also a sound and that provides information about the functions of cochlear outer hair cells responsible for the analyzed frequencies.^21^

In this study, what we considered as the most important were OAE in high frequencies, which qualitatively assess the functional status of outer hair cells in the basal turn of the cochlea. We considered presence or absence of DPOAE.

### Anatomical Assessment

#### Scanning Electron Microscopy (SEM)

**Equipment: Electron Microscope JEOL SCANNING MICROSCOPE - JSM 5200**

Guinea pigs were sacrificed in the scheduled time after administration of drugs by intraperitoneal access and anesthesia with ether, and they were decapitated and the cochleae were removed with the bulla.

To carry out microscopic dissection of cochleae, we performed perfusion with fixation solution of glutaraldehyde at 3% at 4º Celsius and maintained them in the solution for 24 hours for fixation. The following steps were carried out at the Laboratory of Electron Microscopy, Department of Morphology, FMRP-USP:

Through the round window, we injected the fixation solution of glutaraldehyde at 3% in buffer phosphate 0.1 M, pH = 7.4, for 4 hours at 4º Celsius, rinsed three times for 5 minutes with the same buffer. After dissecting the cochlea, it was fixed with osmium tetroxide at 1% for 2 hours at 4º Celsius and submitted to dehydration at room temperature in a growing battery of ethanol (50%, 70%, 90% and 95% - once for 10 minutes in each concentration) and absolute ethanol three times for 15 minutes. When the dehydration was over, we followed it by drying by the critical point method of CO_2_, in which the material was deprived from water. After being fixed in the appropriate specimen holder, the material was recovered in a vacuum chamber with gold vapors and examined under scanning electron microscopy.22., 23., 24., 25.

The results obtained by SEM, after being photographed, were analyzed through cochleograms. We used number counting of outer hair cells in the cochlear turn, in a determined photographic field, and we counted ten cells, present or absent, as shown below in Figure 1.

**Figure 1.1:**
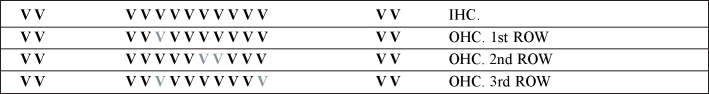
Schematic representation of cochleogram using counting of hair cells by turn, as follows:
**V** = normal hair cell**V** = damaged hair cell

Data were statistically analyzed using the statistical software Statistical Package for Social Sciences - (S.P.S.S.).

We considered the comparison of results and statistically treated only data referring to basal turn, which is the most interesting aspect from the cisplatin damage perspective. However, we showed absolute values, plotted in the graph, with data referring to other cochlear turns.

## RESULTS

**Figure 2:**
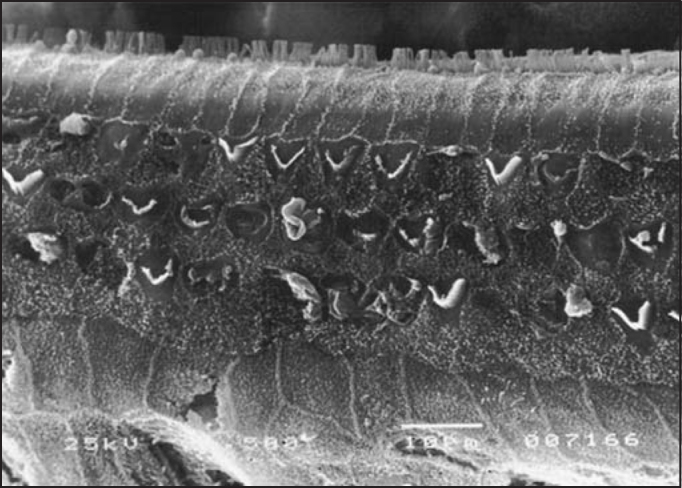
**SEM**, 1,500X magnification, cochlea of guinea pig in group 1.

As to anatomical assessment of group 1, treated with isolated cisplatin (8.0 mg/Kg/day for three consecutive days) there was damage and absence of hairs in the rows of outer hair cells at the level of the basal turn, followed by turns 2 and 3. The most evident affections were seen in the basal turn, but we also observed ciliary distortion with **“v”** pattern disarrangement (**or “w” pattern**), with folded hairs or partial absence of one of the arms of the **“v”** pattern, as in Figure 2. At the level of the inner hair cells, we also observed affections to the hairs, with hairs present but disarranged.

**Figure 3:**
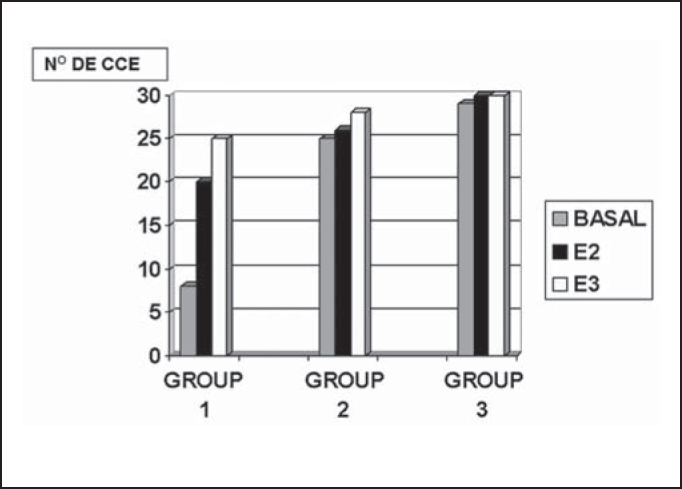
Mean number of outer hair cells present in the cochlear turns - basal, E2 and E3 found in groups 1, 2 and 3.

In Figure 3, we can observe the comparison by stria in cochleae of groups 1, 2 and 3.

In the group previously treated with amifostine, we observed maintenance of normal architecture of outer hair cells, under scanning electron microscopy and distortion product otoacoustic emissions, which were present in all tested cochleae.

As to statistical analysis of presented data, to compare the variable number of outer hair cells in the cochlear basal turn in groups 1, 2 and 3, we used the non-parametric test by Kruskal-Wallis. We decided in favor of a non-parametric methodology because data do not follow the normal course with this variable (Kolmogorov-Smirnoff test with p<0.001). The H0 hypothesis was:

**Figure 4:**
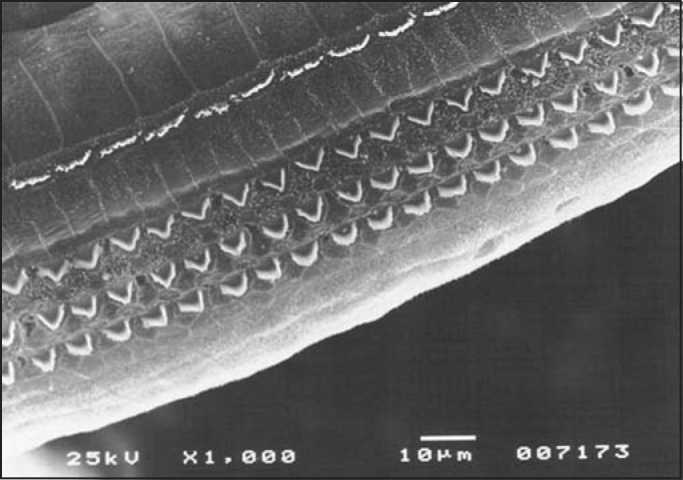
SEM, 1,000X magnification, cochlea of guinea pig in group 2.

**Figure 5:**
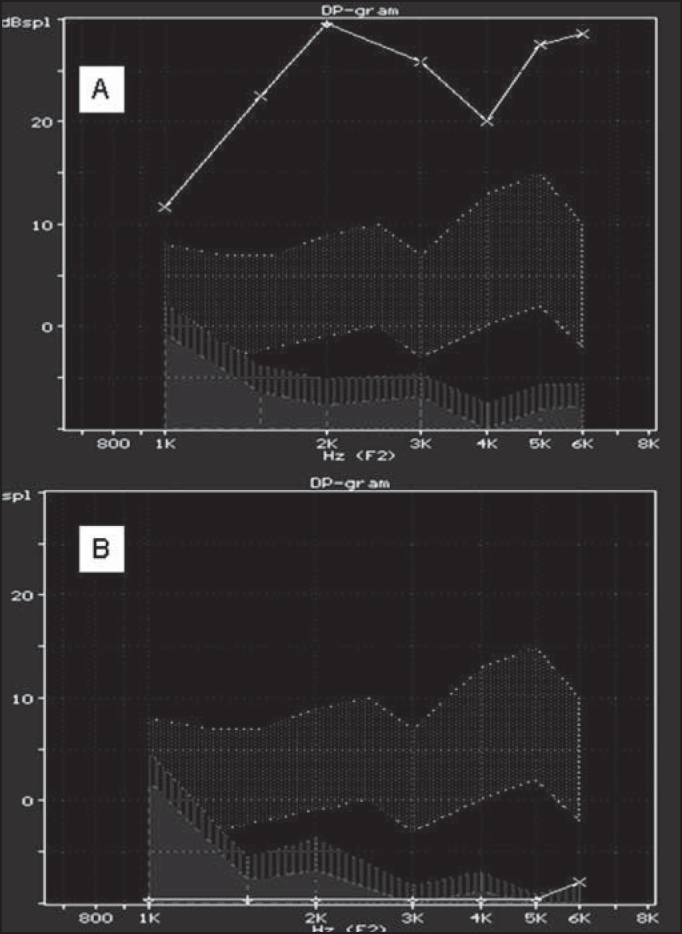
Distortion product otoacoustic emissions in guinea pigs treated with amifostine and cisplatin (group 2) (A) and in guinea pigs treated with cisplatin (group 1) (B).

**H0**: values of 3 groups were equivalent **X**
**H1**: there is at least one pair of groups that differed significantly.

If H0 was rejected, we would use Dunn *post-hoc* test to check which were the detected differences.

We adopted p<0.05 as level of significance. The result of the test was p<0.001, showing that there was significant difference between the groups. Based on Dunn's test, we detected that the number of outer hair cells in Group 1 (CISPLATIN 8.0 mg/Kg/day) was **smaller** than in Group 2 (amifostine 100 mg/Kg/day and CISPLATIN 8.0 mg/Kg/day), which in turn was **equal** to the number in group 3 (amifostine 100 mg/Kg/day).

As to variable DPOAE, we considered the comparison of **presence** and **absence** percentages. In group 1, we reached 100% **absences**, with group of 12, and in groups 2 and 3, we reached 100% of **presence** with groups of 12 and 6. Based on such values shown by Fisher exact test, we reached p<0.001 and groups 2 and 3 were equivalent and significantly different from group 1.

## DISCUSSION

Amifostine has shown low toxicity and good results concerning ototoxic effects of radiotherapy as reported by Foster Nora and Siden, 1997.^13^

Concerning otoprotection against antineoplastic agents, especially cisplatin, Church et al. (1995), found an electrophysiological study of encephalic evoked potential in hamsters, protection by sodium thiosulfate and diethyldimethylthiocarbamate, and they did not observe effective protection against amifostine and fosfomycin^15^.

Kaltenbach et al. (1997) analyzed the same drugs, now associated with anatomical assessment by scanning electron microscopy and brainstem evoked potential. They found 91% maintenance of outer hair cells with sodium thiosulfate, 68% with diethyldimethylcarbamate, 52% with fosfomycin and 45% with amifostine.^16^

In spite of these experimental data in animals, studies in humans with cisplatin for the treatment of different neoplasms have shown a tendency to nephroprotection, neuroprotection, otoprotection and myeloprotection from toxic effects of cisplatin. Foster Nora and Siden, 1997, reported that these studies comprised few subjects with different types of tumors and antineoplastic agent interactions, in addition to receiving different dosages of cisplatin.^13^

Some cancer treatment centers have included in their Guidelines the use of amifostine to prevent some toxic effects of cisplatin, in specific and dose-dependent situations, especially after authorization and standardization of its use as of 1980 by FDA, as provided by the studies by Vincent et al. (2003).^26^

We could observe that, in Albine guinea pigs treated with cisplatin 90 minutes after administration of amifostine, assessed functionally through distortion product otoacoustic emissions and scanning electron microscopy, there was functional and structural significant protection to acute ototoxic effects of cisplatin, different from what was reported by Kaltenbach et al. (1997), in hamsters, showing evidence of the otoprotection potential of amifostine in acute treatment with cisplatin, with doses known to be ototoxic. Such findings may justify their indication of use, as performed in different cancer centers, following the guidelines proposed by FDA (1980).^16^

However, its use is not recommendable in cases of potentially curable tumors because we do not know the exact influence of cisplatin in the efficacy of chemotherapy.12., 14.

## CONCLUSION

Amifostine shows evident signals of otoprotection from ototoxic effects produced by cisplatin in albino guinea pigs.

However, its use is not recommendable in cases of potentially curable tumors because we do not know what is the exact influence of cisplatin in the efficacy of chemotherapy.12., 14.
